# Efficacy and Safety of Sarilumab in COVID-19: A Systematic Review

**DOI:** 10.1155/2021/8903435

**Published:** 2021-10-22

**Authors:** Rajan Chamlagain, Sangam Shah, Basanta Sharma Paudel, Roman Dhital, Bipin Kandel

**Affiliations:** ^1^Tribhuvan University Teaching Hospital, Maharajgunj, Kathmandu 44600, Nepal; ^2^Maharajgunj Medical Campus, Institute of Medicine, Tribhuvan University, Maharajgunj, Kathmandu 44600, Nepal

## Abstract

**Background:**

It has been found that there is overactivation of immune response in patients with COVID-19. Several studies are going on to assess the role of immunomodulation. IL-6 antibodies such as tocilizumab have been found to have efficacy in the treatment of COVID-19. We aim to assess the role of sarilumab in the treatment of COVID-19 through this review. *Main Body*. Functional outcomes were assessed on the basis of PaO_2_/FiO_2_ ratio, mortality, and ventilation. Adverse events of studies were also noted. Five studies were included in the study. There was improvement in PaO_2_/FiO_2_ ratio, reduction in the mortality of the patients, and less number of patients were on ventilation, but there were no significant differences among the comparison and sarilumab group. Sarilumab did not have notable adverse events and can be considered a safe drug.

**Conclusion:**

Sarilumab is a safe drug with good clinical outcomes in patients with COVID-19 and, hence, could be used as an alternative regimen for the treatment. Further prospective studies exploring the relations with baseline biomarkers of inflammation commonly measured such as C-reactive protein and IL-6 would be necessary for a correlation with the treatment.

## 1. Background

**Figure 1:**
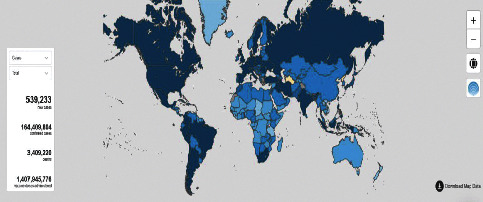
Global spread of COVID-19, showing the number of confirmed cases and death as on May 20 (source: WHO Coronavirus (COVID-19) Dashboard | WHO Coronavirus (COVID-19) Dashboard With Vaccination Data).

The World Health Organization (WHO) has declared COVID-19 as a global pandemic which was first identified in Wuhan, China, in December 2019 [1]. More than 164 million have been infected with more than 3 million deaths worldwide, which is on exponential rise [2] (Figure 1). The range of symptoms of COVID-19 varies from asymptomatic to pneumonia-like symptoms and may even lead to multiorgan failure and death. As per the Centers for Disease Control and Prevention (CDC), the clinical manifestations of COVID-19 includes fever or chills, cough, shortness of breath or difficulty breathing, muscle or body aches, fatigue, headache, sore throat, a new loss of taste or smell, congestion or runny nose, nausea or vomiting, and diarrhea [3]. Double B.1.617 (E484K and L452R) and triple mutant (V382L) strains of single-stranded RNA virus have been reported from twenty-one nations around the globe, and these patients have presented with increased severity of symptoms as compared to previous strains and have been classified as variants of concern by the World Health Organization [4].

It is assumed that COVID-19 results in Cytokine Release Syndrome (CRS), which leads to dysregulation and excessive increase in immune response of the host even though the exact mechanism remains unknown [5]. Many proinflammatory cytokines are involved in CRS, and among them, Interleukin-6 (IL-6) is the most important. Increase in the circulating level of proinflammatory cytokines (IL-1 and IL-6) and chemokines results in adverse clinical outcomes of which increasing IL-6 is a poor prognostic factor [6].

There is no definitive treatment for this condition, and hence, symptomatic management is the only treatment so far. The Food and Drug Administration (FDA) has approved antiviral drug (remdesivir), corticosteroid (dexamethasone, prednisolone, and methylprednisolone), and monoclonal antibodies, particularly IL-6 antibodies (tocilizumab, siltuximab, and anakinra), for the treatment of COVID-19.

Sarilumab, initially approved for use in rheumatoid arthritis, is a human monoclonal antibody which acts against IL-6 receptors. Additionally, this drug has been considered for off-label use in the treatment of COVID-19. Recent trials have reported that it reduces the duration of hospital stay, and it has been considered as a safe drug. There is no concrete evidence among the trials to have its efficacious role in the treatment of COVID-19. To our knowledge, no systematic review is available to assess the role of sarilumab. In this review, we aim to analyze the role of the immunomodulator, sarilumab, in the treatment of COVID-19 patients.

## 2. Methodology

### 2.1. Study Identification

The systematic search was conducted in online databases PubMed, https://clinicaltrials.gov, and EMBASE using the keywords “Sarilumab,” “COVID,” and “SARS COV-2,” connected with “OR” and “AND” Boolean operators, while the article search was conducted from 2018 onwards. The reference list of included trials and articles were also searched to identify additional articles. The abstract was screened using microsoft excel 2013 (windows version) followed by full-text screening. For those articles with partial information, concerned authors were contacted via e-mail. For future analysis, we retrieved all the references in all manuscripts. Preferred Reporting Items for Systematic Reviews and Meta-analysis (PRISMA) [7] and protocol for reviews detailed in the Cochrane Handbook for Systematic Reviews of Interventions [8] were followed for reporting the article. The first active search was performed on 2 March 2021, while the last was performed on 10 March 2021.

### 2.2. Inclusion Criteria

Studies were selected on the basis of the following criteria:

- Patients with COVID-19, including all its stages
- Any study design (randomized controlled trials with double-blindfold/observational studies/case-control studies) on humans reporting clinical outcome on sarilumab
- Patients who received sarilumab as an experimental drug

### 2.3. Exclusion Criteria

Studies were excluded on the basis of the following criteria.

- Study on other experimental animals
- Trials which did not use sarilumab as an experimental drug
- Articles that were not available in English
- All other forms of articles such as case reports

### 2.4. Data Extraction

Two authors (SS and RC) extracted the data from the included studies, and the studies and were recorded as follows:

  A) Author; (B) year of study; (C) stage of the trial; (D) sample size; (E) study design; (F) country of study; (G) mean age; (H) gender; (I) efficacy measures; (J) adverse reactions; and (K) dose of sarilumab

Adverse reaction was recorded for all the studies included. Extracted data were checked by another author (BSP), and disagreement was resolved by discussion with other authors (RD and BK).

### 2.5. Quality Assessment

We evaluated the following items in the assessment: (1) clarity of the study objectives; (2) whether the study period (start date and end date) was stated clearly; (3) whether the description of the patient selection criteria was clear or not; (4) study was conducted in multicenter or not; (5) the stated sarilumab treatment method and dose; (6) whether the baseline equivalence groups were clearly considered; (7) the definition of the primary outcome (PaO_2_/FiO_2_ ratio or overall mortality or ventilation requirement) prior to the study; (8) if the follow-up period was long enough (months); (9) whether a clear hazard ratio (HR) with 95% confidence intervals (95% CI) was stated; and (10) the limitations of each study were considered. We did not use quality assessment as exclusion criteria. According to the quality items used in each study (score range 0–10), the papers were assessed.

### 2.6. Efficacy Measurement

The functional outcomes of patients were analyzed under the following subheadings:

- Partial pressure of arterial oxygen (PaO_2_)/fraction of inspired oxygen (FiO_2_) ratio: it is the ratio of arterial oxygen partial pressure to fractional inspired oxygen. It helps to determine how well oxygen is moving from the lungs to blood [9].
- Mortality: mortality was determined by using different scales such as the six-category/seven-category ordinal scale.
- Need of mechanical ventilation (noninvasive ventilation (NIV) and invasive mechanical ventilation (MV)).

### 2.7. Data Synthesis

All identified studies were included in the narrative summary with summary tables for characteristics. In addition, data were summarized using descriptive statistics. We used means for continuous variables and frequencies and percentages for dichotomous variables.

## 3. Results

### 3.1. Literature Search

**Figure 2:**
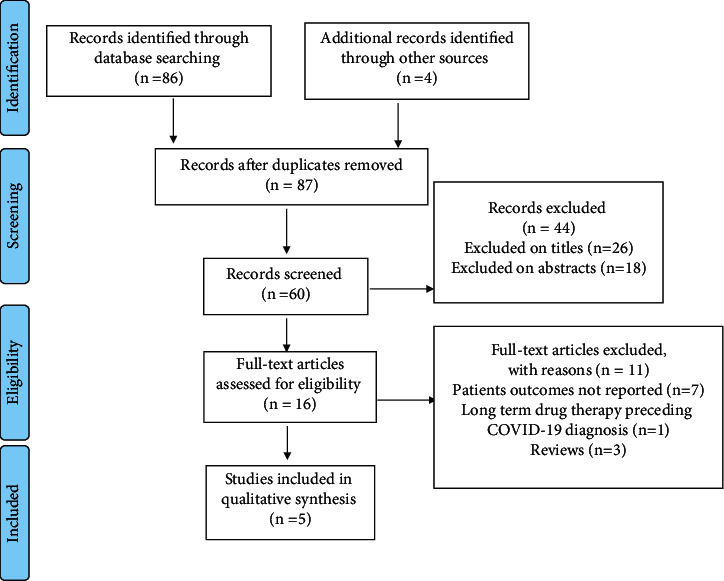
PRISMA guidelines for article identification and selection.

The literature search resulted in 90 studies. After the complete screening process of titles, abstracts, and full texts, 85 studies did not meet the eligibility criteria. Finally, five articles with different study designs that met the criteria were included in the review. A description of study selection is shown in the PRISMA flow diagram in Figure 2.

### 3.2. Quality Assessment Results

With the use of the quality assessment tool, individual questions of the study were answered in “yes” or “no” with 1 point for “yes” and no points for “no.” The total score was added for the individual study as denoted in the respective columns. The quality of the five included studies was fair with an average quality score of 6.6 and a median score of 7 (range 3–10) (Table 1).

### 3.3. Literature Identification

Among the included studies, four were conducted in Italy. The study by Lescure et al. was a multinational and randomized, double-blind, placebo-controlled phase III trial. The study by Montesarchio et al. was retrospective. The other three studies were open-label observational studies. The study duration, sample size, efficacy measures, adverse reactions, and dose of sarilumab are indicated in Table 2.

### 3.4. Patient Characteristics

A total of 552 patients were included from five studies of which 370 (67.0%) were males. The mean age of the included patients was 60.4 years.

### 3.5. Measurement of Efficacy

#### 3.5.1. Partial Pressure of Arterial Oxygen (PaO_2_)/Fraction of Inspired Oxygen (FiO_2_) Ratio

Clinical improvement with shorter duration was observed in 91% cases with PaO_2_/FiO_2_ ratio >100 mm Hg, while the clinical improvement with longer duration was observed in 41% cases with PaO_2_/FiO_2_ ratio <100 mm Hg (7 (5–15) days vs. 28 (18–28) days; HR 0.18; 95% CI 0.02–0.26; *P*=0.0001) [10]. The median PaO_2_/FiO_2_ at baseline for all patients was 122 (range 83–240), and 8 of 15 (53.3%) were intubated at the time of administration of sarilumab [11]. The median baseline PaO_2_/FiO_2_ for patients who responded to sarilumab was 121 mm of Hg. The median baseline PaO_2_/FiO_2_ was 178 mm of Hg in patients who did not respond to sarilumab [11].

There was improvement in oxygenation as indicated by the Horovitz index by ≥ 50 for 48 hours [12]. PaO_2_/FiO_2_ was associated with clinical improvement in both univariate and multivariate analysis and was found to be an independent predictor for clinical improvement in multivariate analysis (HR: 0.87; 95% CI 0.86 to 0.99; *P*=0.03) [10]. There was a negative insignificant correlation (*r* = −0.0005 and *P*=0.98) between PaO_2_/FiO_2_ ratio and lung consolidation [10].

In the medical ward, 30 (85.7%) no longer needed oxygen supplementation and PaO_2_/FiO_2_ ratio was increased in patients with positive SARS-CoV-2 after infusion of sarilumab (3(8.8%) at FiO2 24%, 1 (2.8%) at FiO2 35%, and 1(2.8%) at HFNC (FiO2 35%)), while in the Intensive Care Unit (ICU), 8 (88.9%) patients were shifted to medical wards (4 of them discharged), 1 (11.1%) was in HFNC (FiO_2_ 26%), and 5 (35.7%) patients were still in the ICU [13].

#### 3.5.2. Mortality

Median time to death was longer in the sarilumab group (19 days, interquartile range (IQR) (13–26) vs. 4 days, IQR (3-4); *P*=0.006) [10]. One patient who had no improvement in Horovitz's functional index died after 13 days of hospitalization [12]. 11% (44 of 416) of patients died due to treatment-emergent adverse events, with similar rates between the treatment group (placebo (11%); sarilumab 200 mg (11%), and sarilumab 400 mg (10%)) [14]. Two patients died in the ICU, not attributable to sarilumab. Overall mortality rate was 5.7% after administration of sarilumab: 1 (2.5%) patient died in the medical ward, and 2(14.2%) patients died in the ICU, respectively [13]. Five patients who were intubated or on ventilators received sarilumab later died after the treatment [11].

#### 3.5.3. Ventilation

Six of 28 patients of the sarilumab group and 7 of 28 patients of the control group (*P*=0.99) were on mechanical ventilation [10]. Among eight patients who required mechanical ventilation prior to administration of sarilumab, pulmonary function was improved in 3 of them and 1 died after being extubated due to a massive embolism [11]. The details of invasive and noninvasive mechanical ventilation in study by Gremese et al. are shown in Table 3.

### 3.6. Adverse Effects

In a study by Montesarchio et al., 1 patient developed grade II thrombocytopenia [11]. 12/28 patients (43%) in the sarilumab group and 10/28 patients (36%) in the control group reported adverse events between baseline and day 28. Six patients (21%) in the sarilumab group and five (18%) in the control group had bacterial infections in the ICU with polymicrobial infection in 4/6 cases in the sarilumab group [10]. The rates of treatment-emergent adverse events, infection, and treatment-emergent adverse events leading to death were similar among the treatment groups [14].

## 4. Discussion

An IL-6 receptor antibody, tocilizumab, initially approved for rheumatoid arthritis has been approved for off-label use against COVID-19. Likewise, sarilumab, another IL-6 antibody, is also under trials for the treatment of COVID-19. There was improvement in the respiratory function as measured by PaO_2_/FiO_2_ ratio with the use of sarilumab [10, 11]. But, the improvement in PaO_2_/FiO_2_ ratio was not statistically significant with the comparison group. Tocilizumab has already been established as an effective drug in improving oxygen transfer from lungs to blood [15]. Mortality assessment on a 6- or 7-point ordinal scale determined that the number of deaths was decreased but it was not statistically significant among the sarilumab group and comparison group [13, 14]. The probable reason for these could be concomitant the use of other medications such as dexamethasone, lopinavir, and heparin. Similar to sarilumab, lopinavir also reported similar mortality rates among lopinavir and standard care groups [16]. Furthermore, dexamethasone reduced the death by one-third and one-fifth among COVID-19 patients who were on mechanical ventilation and without invasive mechanical ventilation, respectively, in a RECOVERY trial [17]. But, the same trial did not report a reduction in mortality for the patients who were not on respiratory support [17]. Early administration of cytokine modulation therapy (Tocilizumab), before the onset of symptoms, deteriorates the onset of cytokine storm. Hence, the timing of IL-6 therapy may also have affected the mortality.

Immunomodulation is thought to be beneficial in serious cases of COVID-19. When sarilumab was given in patients with severe COVID-19 in the ICU, it was found to have significant improvement in health condition, and they were transferred to medical wards [13]. Suppression of IL-6 alone may not be sufficient to explain the inflammatory phase of this disease because of the involvement of other inflammatory cytokines and chemokine. Tocilizumab found no significant improvement in reducing disease severity and time to intubation or death in a randomized controlled trial [18]. Similar findings were observed in a trial of dexamethasone versus usual care in patients with COVID-19; this trial suggested survival benefits that were associated with the intensity of respiratory support [17]. Sarilumab may reduce the number of days of hospitalization. Less number of patients went on mechanical ventilation or death in EMPACTA, randomized placebo-controlled study for tocilizumab [19].

Sarilumab is a safe drug with less treatment-emergent adverse effects. Most of the studies did not report the adverse effects, and among the studies that reported adverse effects, treatment-emergent adverse events were not available. Tocilizumab, another IL-6 antibody, was found to be safe, but it showed an increase in the incidence of adverse infectious events such as bacterial and fungal infections with no better outcomes with 400 mg dose compared with patients treated with a single dose of tocilizumab 400 mg [20]. The included studies were conducted for a shorter duration, and hence, long-term adverse events were not available.

Our review had several limitations. Firstly, patients included in most of the studies were not recruited on clinical markers of inflammation or worse prognosis. Concomitant use of other therapies along with sarilumab also had interfered with the outcomes. Efficacy endpoints that were used to assess may be insensitive for a wide range of COVID-19 because its symptom ranges from asymptomatic to respiratory failure. Also, the functional scale to assess mortality was different across different studies. The sample sizes of the included studies were small, and the duration of the study was also short. Finally, most of the studies were open label, were not randomized, and were conducted in a single-center setting.

## 5. Conclusions

Sarilumab is a safe drug with good clinical outcomes in patients with COVID-19 and, hence, could be an alternative regimen for the treatment. Further prospective studies with larger sample size and long-term follow-up are required to assess the efficacious role of the IL-6 antagonist. Exploring relations with baseline biomarkers of inflammation such as C-reactive protein (CRP) and IL-6 would be necessary for a correlation with the treatment.

## Figures and Tables

**Table 1:** Quality assessment of the included studies.

Criteria	Studies				
	Della-Torre et.al.	Montesarchio et.al.	Benucci et.al.	Lescure et.al.	Gremese et.al.
Clarity of the study objectives	Yes	Yes	Yes	Yes	Yes
Whether the study period (start date and end date) was stated clearly or not	Yes	Yes	Yes	Yes	Yes
Whether the description of the patient selection criteria was clear or not	Yes	Yes	No	Yes	Yes
The stated sarilumab treatment method and dose	Yes	Yes	Yes	Yes	Yes
Study was conducted in multicenter or not	No	No	No	Yes	No
Whether the baseline equivalence groups were clearly considered or not	Yes	No	No	Yes	Yes
The definition of the primary outcome (PaO_2_/FiO_2_ ratio or overall mortality or ventilation requirement) prior to the study	Yes	Yes	No	Yes	Yes
If the follow-up period was long enough (at least two months)	No	No	No	Yes	No
Whether a clear hazard ratio (HR) with 95% confidence intervals (95% CI) was stated or not	No	No	No	Yes	No
The limitations of each study were considered	Yes	Yes	No	Yes	Yes
Overall score	7	6	3	10	7

Mean: 6.6.

**Table 2:** Details of the included studies in this review.

Author	Study duration	Stage of trial	Sample size	Study design	Country of study	Mean age	Gender (male)	Efficacy measures	Adverse reactions	Sarilumab dose
Della-Torre et.al.	14 March–2 April, 2020		56	Open-label observational study	Italy	56 (51–60)	44	PaO_2_/FiO_2_ ratio Mortality (six-point scale) Others	Infections Neutropenia Increase of liver enzymes Thromboembolism	Single IV 400 mg Two single-dose 200 mg sarilumab IV over 1 hour
Montesarchio et.al.	March 26, 2020, to April 3, 2020		15	Retrospective	Italy	59 (53–75)	12	PaO_2_/FiO_2_ ratio Mortality Others	Thrombocytopenia	Single IV 400 mg Two doses (2^nd^) after 24 hours of the first
Benucci et.al.	14 days		8	Open-label, observational study	Italy	62	6	PaO_2_/FiO_2_ ratio Others	NA	Single IV 400 mg Two single-dose 200 mg sarilumab IV over 1 hour
Lescure et.al.	60 days	Phase III	420	Randomised, double-blind, placebo-controlled study	Multinational	59 (50–68)	261	PaO_2_/FiO_2_ ratio Mortality (seven-point scale) Others	Infection Treatment-emergent adverse effect	Sarilumab 200 or 400 mg IV
Gremese et.al.	March 23rd till April 4, 2020		53	Open-label, observational study	Italy	66 (40–95)	47	PaO_2_/FiO_2_ ratio Mortality Others	No notable adverse effects	400 mg IV on day 1 and 2; sarilumab 200 mg IV

NA: not available; IV: intravenous.

**Table 3:** Functional efficacious outcomes of the included studies.

Author	Efficacy outcomes	Description, *n* (%)
Della-Torre et.al.	PaO_2_/FiO_2_ ratio; *n* (%)	**200–300 mm of Hg**
		Total: 4 (7)
		Sarilumab: 1 (4)
		Comparison: 3 (11)
		*P* value: 0.61
		**100–200 mm of Hg**
		Total: 22 (39)
		Sarilumab: 10 (36)
		Comparison: 12 (43)
		*P* value: 0.78
		**<100 mm of Hg**
		Total: 30 (54)
		Sarilumab: 17 (60)
		Comparison: 13 (46)
		*P* value: 0.42
	Mortality; *n* (%)	Sarilumab: 2 (7)
		Comparison: 5 (18)
		*P* value: 0.42
	Clinical improvement; *n* (%)	Sarilumab: 17 (60)
		Comparison: 18 (64)
		*P* value: 0.99
	Mechanical ventilation; *n* (%)	Sarilumab: 6 (21)
		Comparison: 7 (25)
		*P* value: 0.99
Montesarchio et.al.	PaO_2_/FiO_2_ ratio	122 (83–240)
	Mortality; *n* (%)	5 (33.3)
	Intubated vs. not intubated; *n* (%)	8 (53.3) vs. 7 (46.7)
Benucci et.al.	PaO_2_/FiO_2_ ratio	Improvement of oxygenation expressed by an increased SpO_2_/FiO_2_ ratio (Horovitz index) by 50 or higher compared to nadir SpO_2_/FiO_2_ for at least 48 hours: seven of them showed an improvement of the Horovitz index
	Mortality; *n*	7 survived and 1 died
Lescure et.al.	PaO_2_/FiO_2_ ratio	Total: 237·5 (173·6–300·0)
		Placebo: 240·0 (190·0–332·1)
		Sarilumab (200 mg): 230·0 (165·0–296·9)
		Sarilumab (400 mg): 237·5 (172·7–293·8)
	Mortality (seven-point scale); *n* (%)	Total: 9 (11%)
		Sarilumab (200 mg): 17 (11%)
		Sarilumab (400 mg): 18 (10%)
	Noninvasive ventilation; *n* (%)	Total: 7 (2%)
		Placebo: 2 (2%)
		Sarilumab (200 mg): 3 (2%)
		Sarilumab (400 mg): 2 (1%)
	Invasive mechanical ventilation; *n* (%)	Total: 48 (12%)
		Placebo: 9 (11%)
		Sarilumab (200 mg): 16 (10%)
		Sarilumab (400 mg): 23 (13%)
Gremese et.al.	PaO_2_/FiO_2_ ratio	No ICU care: 167.5 (125.4–226.5)
		ICU care: 101.0 (89.0–141.0)
		*P* value: 0.007
	Mortality; *n* (%)	No ICU care: 1 (2.5%)
		ICU care: 2 (14.2%)

ICU: intensive care unit.

## Data Availability

Qualified researchers may request access of the quality assessment table from the corresponding author.

## Abbreviations

COVID-19: Coronavirus disease 2019
IL: Interleukin
CRP: C-reactive protein
ICU: Intensive care unit
HR: Hazard ratio
HFNC: High-flow nasal canula
PaO_2_/FiO_2_: Partial pressure of arterial oxygen/fraction of inspired oxygen
WHO: World Health Organization
PRISMA: Preferred Reporting Items for Systematic Reviews and Meta-Analysis
CDC: Centers for Disease Control and Prevention
FDA: Food and Drug Administration
IQR: Interquartile range
NIV: Noninvasive ventilation
MV: Mechanical ventilation.
